# Developing Super-Hydrophobic and Abrasion-Resistant Wool Fabrics Using Low-Pressure Hexafluoroethane Plasma Treatment

**DOI:** 10.3390/ma14123228

**Published:** 2021-06-11

**Authors:** Shama Parveen, Sohel Rana, Parikshit Goswami

**Affiliations:** Technical Textiles Research Centre, Department of Fashion and Textiles, University of Huddersfield, Queensgate, Huddersfield HD1 3DH, UK; S.Rana@hud.ac.uk (S.R.); P.Goswami@hud.ac.uk (P.G.)

**Keywords:** wool fibres, hexafluoroethane plasma, abrasion resistance, water contact angle, dyeing behaviour

## Abstract

The growing interest in wool fibres as an eco-friendly and sustainable material for diverse industrial applications requires an enhancement of their functional performance. To address this, wool fabrics were treated in the present research with low-pressure hexafluoroethane (C_2_F_6_) plasma to impart superhydrophobicity and improve their abrasion resistance. Unscoured and scoured wool fabrics were treated with C_2_F_6_ while varying plasma power (80 W and 150 W), gas flow rate (12 sccm and 50 sccm) and treatment time (6 min and 20 min), and the effect of plasma parameters on the abrasion resistance, water contact angle and dyeing behaviour of the wool fabrics was studied. Martindale abrasion testing showed that the surface abrasion of the wool fabrics increased with the number of abrasion cycles, and the samples treated with 150 W, 20 min, 12 sccm showed superior abrasion resistance. The scoured wool fabrics showed a contact angle of ~124°, which was stable for only 4 min 40 s, whereas the plasma-treated samples showed a stable contact angle of over 150°, exhibiting a stable superhydrophobic behaviour. The C_2_F_6_ plasma treatment also significantly reduced the exhaustion of an acid dye by wool fabrics. The EDX study confirmed the deposition of fluorine-containing elements on the wool fabrics significantly altering their properties.

## 1. Introduction

The textile industry is one of the largest manufacturing industries, with a high impact on the economy and ecosystem of the globe [1]. Currently, the application of textiles is not restricted only to the apparel and fashion industries, but has entered extensively into numerous other industrial sectors including medical, sports, transportation, architecture, and civil construction, among others [1]. Due to the widespread use of textiles in different fields, a huge pressure exists in the current textile industry to modify the traditional processes in order to reduce their adverse effects on the environment. Textile processing involves the release of toxic, hazardous, and non-biodegradable wastes, which has become a serious concern for many countries with stringent environmental protection acts [1]. Clean and dry treatments are preferred over the wet processing of textiles to minimise the amount of pollutants released into the environment [1]. Researchers are finding alternative approaches to reduce the use of obnoxious techniques in textile processing, and one of the emerging alternatives is plasma technology [2]. Plasma technology has garnered much attention due to its potential applications in other industrial sectors such as the medical, electronics, automotive, aerospace etc. [2,3]. In textile manufacturing, plasma technology has already been explored to produce hydrophilic or hydrophobic/oleophilic finishing on fabric surfaces [3,4]. In most cases, the plasma finishing provided a unique set of properties which could not be achieved easily by wet treatments, due to the huge flexibility in the use of different monomers/gases in the plasma process [3,4]. Additionally, the surface modification achieved by plasma does not alter the bulk textile properties, making this technology highly suitable for the textile industries [3,4].

Plasma processes for textiles can be classified as either atmospheric or low-pressure plasma [4,5,6]. Plasma treatment of textiles may lead to chain scission on the fibre surfaces and to surface etching, cleaning or activation, and this can be obtained using non-polymerising gases such as helium, oxygen, air and nitrogen [4,5,6]. Plasma treatment may also result in polymerisation or grafting as observed in case of various polymerising gases and precursors, e.g., fluorocarbons, hydrocarbons and silicon containing monomers [4,5,6]. In the past, it was believed that only low-pressure plasma could provide uniformity, reproducibility, use of various reagent gases, and selective modification of surfaces. However, due to recent advances in technology, atmospheric plasma can also achieve the above characteristics for textiles, although modifications to machines and processes are still required to achieve widespread application [4,5,6]. Until now, both forms of plasma technologies have been explored to (i) clean the surfaces to change the wettability and surface texture of materials, (ii) generate polymers on the surfaces with the desired properties, (iii) generate free radicals to promote graft polymerisation for producing hydrophobic or hydrophilic surfaces, and (iv) increase micro/nano roughness, which improves the adhesion of finishing agents and influences anti-felting finishing [4,5,6].

The present research work is focused on the development of hydrophobic surfaces on wool fabrics using C_2_F_6_ gas in a low-pressure plasma chamber and improving other physical properties such as abrasion resistance. Hydrophobic textiles have been developed by various researchers for self-cleaning, for water–oil separation, and for numerous biomedical applications [6,7,8]. Generally, the hydrophobic treatment of textiles involves wet chemical processes in which fabrics are commonly coated or graft-copolymerised with long-chain hydrocarbons, silicone compounds, and nanomaterials [9,10,11]. More recently, bio-mimicking of the lotus effect on textile surfaces to considerably reduce the surface energy and achieve super-hydrophobicity has gained a lot of attention [7,10]. For this purpose, researchers have developed rough hierarchical textures on substrates using different methods, including ultrasonic irradiation [11], hydrothermal processes [12], thermal curing [13] etc. These techniques involve high energy consumption and use of inorganic or non-metallic nanomaterials (e.g. SiO_2_, TiO_2_, carbon nanotubes, ZnO etc.), which are hazardous and have toxicity issues. To overcome these drawbacks, a cost-effective, environmentally friendly and scalable approach is highly sought after, and plasma technology is emerging as a clean, green and scalable surface modification technique in textiles to improve hydrophobicity and impart a wide range of other finishes.

Although plasma technology has been used for different textile fibres, studies on introducing hydrophobicity into wool fibres through plasma treatments are very rare. This could be due to the fact that wool fibres inherently present certain levels of hydrophobicity, and looking at the existing applications of woollen textiles in the clothing sector, attempts have been made rather to improve their hydrophilicity to facilitate moisture sorption and dyeing behaviour. In a recent study performed by Sun et al., wool fabrics were treated with low-temperature plasma (13.56 MHz RF plasma) using C_2_F_6_ gas for 3 min at a power of 80 W and a flow rate of 0.1 dm^3^ min^−1^. It was observed that the C_2_F_6_ plasma treatment significantly reduced the roughness and improved the hydrophobicity of wool fibres [14]. Looking at the extensive use of wool fibres in the apparel and fashion sectors, as well as in other emerging applications in the technical textile area, increased demand can be noticed for the clean and dry hydrophobic finishing of wool fibres (to prevent easy wetting, soiling and mould formation in the woollen textiles), and C_2_F_6_ plasma treatment could be advantageously used for this purpose. Therefore, further in-depth investigation is essential to understand the influence of C_2_F_6_ plasma treatment on the hydrophobicity and other properties of wool fibres, which has been addressed in this research work.

Most technical and apparel applications demand textiles with good abrasion resistance. As wool fibre textiles have a moderate abrasion resistance [15], the abrasion resistance of wool fabrics has been improved by blending with nylon and elastic fibres [16], tailoring yarn and fabric structures (e.g., yarn structure, twist, count, hairiness, fabric weave, thread density, interlacements, etc.) [15,17,18], as well as using nano finishing [18]. Although fluoropolymer coatings are known to have high abrasion resistance, no attempts have been made to date to improve abrasion resistance of wool fabrics using fluoropolymer coatings or deposition of fluoropolymers through plasma treatment. In the present study, therefore, the effect of C_2_F_6_ plasma treatment on the abrasion resistance of wool fabrics has also been investigated, along with hydrophobicity and dyeing behaviour.

## 2. Experimental Section

### 2.1. Raw Materials

**Figure 1:**
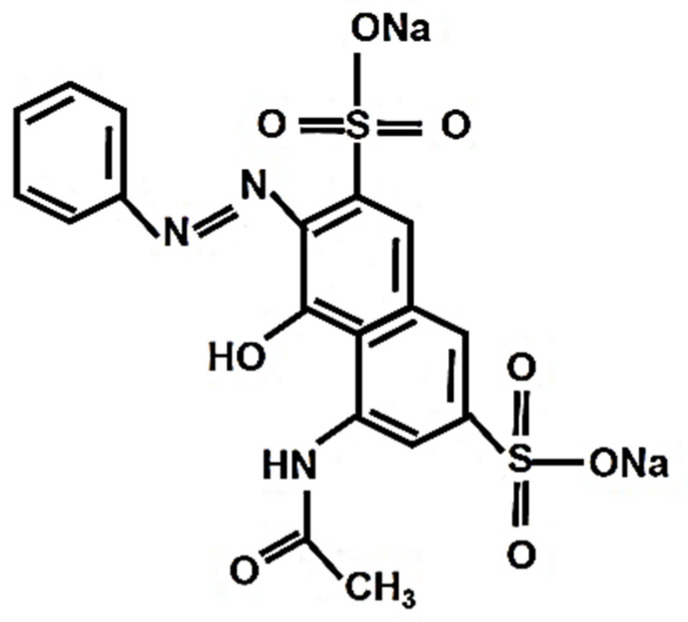
Chemical structure of CI Acid Red 1 dye (https://www.sigmaaldrich.com, Accessed Date: 21 February 2019).

Wool fabrics with an areal density of 475 g m^−2^ (2/2 broken twill, picks per inch: 34, ends per inch: 31, warp crimp: 15%, weft crimp: 28%) were purchased from Whaley Bradford Ltd., UK. The scouring agent, Ultravon JUN (a non-ionic detergent) was purchased from Huntsman Textile Effects, GmbH (Langweid am Lech, Germany) and sodium carbonate was supplied by Intra Laboratories (Plymouth, UK). The C_2_F_6_ cylinder for plasma treatment was purchased from BOC, UK. CI Acid Red 1 dye (dye content: 60%) was purchased from Sigma Aldrich (Gillingham, UK). The chemical structure of this dye is shown in Figure 1, and its properties are listed in Table 1.

### 2.2. Scouring of Wool Fabrics

Wool fabrics were scoured with 1 g dm^−3^ Ultravon JUN nonionic detergent and 2 g dm^−3^ sodium carbonate in a 5 dm^3^ metal container using a material-to-liquor ratio of 1:20 for 15 min at 60 °C. The washed fabrics were line dried for 48 h and then kept in the conditioning laboratory (temperature of 20 ± 2 °C and relative humidity of 65 ± 2%). Prior to the plasma treatment, the samples were oven dried at 70 °C for 24 h and then stored in a desiccator.

### 2.3. Plasma Treatment of Wool Fabrics

Both unscoured and scoured wool fabrics were treated with C_2_F_6_ plasma under four different conditions. Plasma power, gas flow rate, and treatment time were varied, as listed in Table 2. The plasma treatment was carried out in a PICO low-temperature, low-pressure plasma machine (Pico-AR-200-PC-c, Diener GmbH, Ebhausen, Germany). Plasma was generated using a radio frequency (RF) plasma generator with a frequency of 13.56 MHz. The samples were placed in a vacuum chamber over a flat stainless steel sample holder (carrier) kept at a distance of 8.5 cm from the electrode. Before introducing the C_2_F_6_ gas into the plasma chamber, a vacuum was created by removing air with the help of a vacuum pump. The samples were exposed to C_2_F_6_ plasma on both top and bottom surfaces. To ensure plasma treatment on both surfaces, the samples were first treated with plasma for a specific time period (as provided in Table 2) and then placed upside down on the sample holder to expose the bottom surface and treated again for the same time period.

### 2.4. Characterisation of Abrasion Resistance

After the plasma treatment, the samples were characterised for abrasion resistance in a Martindale tester following the BS EN ISO 12947-3:1998 standard (using a diameter of aberrant felt underlay of 140 mm, a diameter of underlay foam backing and specimen of 38 mm, and an abrasion load of 795 ± 7 g). In this test, under the specified abrasion loading, the aberrant felt was rubbed across the wool fabric sample’s surface and the degree of abrasive wear was checked periodically from the visible damage and weight loss of the test fabrics. The samples were tested up to 10,000 abrasion cycles, which did not lead to failure of the samples. The visible damage on the fabric surface was analysed from pictures taken using an Olympus Camera (OM-D E-M10 Mark II digital camera fitted with an Olympus M.Zuiko Digital ED 60 mm F2.8 Macro lens, Southend-on-Sea, UK) and the weight loss of the samples after each 500 cycles was determined using a digital microbalance.

### 2.5. Energy Dispersive X-ray (EDX) Analysis

Elements present in wool fibre surface before and after plasma treatment were characterised using EDX analysis using Si(Li) detector and an acceleration voltage of 5 kV. EDX analysis was performed in a FEG250 SEM instrument (FEI Europe, Eindhoven, The Netherland) under high vacuum. For this analysis, the samples were placed onto 32 mm aluminium stubs and adhered using a piece of double-sided adhesive carbon tape (Agar Scientific, Stansted, UK). Prior to the EDX analysis, the samples were coated with approximately 30 nm gold using a Quorum SC7620 Sputter Coater (East Sussex, UK) via a Gold-Palladium disc in an argon atmosphere, to avoid charging of the samples during the measurement. The peaks due to gold coating were eliminated from the spectra later on using EDX software to nullify its effect on the elemental measurement.

### 2.6. Measurement of Contact Angle

Prior to the image capturing, all samples were placed in the conditioning room (temperature of 20 ± 2 °C and relative humidity of 65 ± 2%) for 48 h. The samples with 5 cm × 2 cm dimension were placed on a table top and a camera (Olympus OM-D E-M10 Mark II digital camera fitted with an Olympus M.Zuiko Digital ED 60 mm F2.8 Macro lens) was placed in the horizontal mode on a tripod stand at a fixed height with respect to the samples. The distance between the tip of the used micropipette and the sample surface was kept constant at 2 cm. To measure the static contact angle of a sample, a water droplet from the 200 µL tip of the micropipette was dropped onto the sample surface vertically, and the static contact angle was measured using Image J software (64 bit Java 1.8.0_172) within 10 s of the initial contact of the drop. Five measurements were made for the static contact angle at different locations of the specimen, and the average value is reported. To study the change in contact angle with time, five videos (each for 20 min) were taken for each plasma-treated and untreated fabric samples.

### 2.7. Dyeing of Wool Fabrics

Unscoured, scoured and plasma-treated wool fabrics (both scoured and unscoured) were dyed with CI Acid Red 1 dye at 2% on the mass of fibre (omf) in a Roaches Pyrotec IR Dyeing machine using individually sealed dye tubes, with a liquor ratio of 25:1. Sulphuric acid was used to maintain the pH at 2.5 and 5% sodium sulphate (anionic levelling agent) was added to the dye bath in the beginning of dyeing. Temperature increased at a rate of 2 °C min^−1^ to 90 °C, and then kept constant for further 60 min. Next, the dye tubes were cooled down to 40 °C at a rate of 2 °C min^−1^ and then the samples were removed from the dye tubes.

### 2.8. Measurement of Dye Uptake and Color Strength

The left-over dye solutions inside the dye tubes were collected after the dyeing process and analysed to measure the concentrations of dye remaining in the dye bath in the case of both untreated and plasma-treated samples. The dye exhaustion, i.e., the percentage of dye taken up by the samples (with respect to the original dye concentration in the dye bath), was then calculated. The dye concentration in the solutions was measured using UV-Vis spectroscopy (Jasco V-730 spectrophotometer, Easton, UK). The absorbance was measured in the range of 400–700 nm with an interval of 1 nm and the absorbance at 532 nm ($\lambda_{max}$) was recorded. A calibration curve for absorbance vs. dye concentration was plotted (by measuring the absorbance of known dye solutions), and the dye concentrations in the dye tubes were then calculated using this calibration curve.

The colour strength, i.e., K/S values (where K is the absorbance coefficient and S is the scattering coefficient) were measured using a Datacolor 650 colour spectrometer (Datacolor, Lawrenceville, NJ, USA) at 20 nm intervals and the K/S values at *λ_max_* were reported. Three measurements were performed for each sample and the average value has been reported.

## 3. Results and Discussion

### 3.1. Effect of Plasma Treatment on Abrasion Resistance

**Figure 2:**
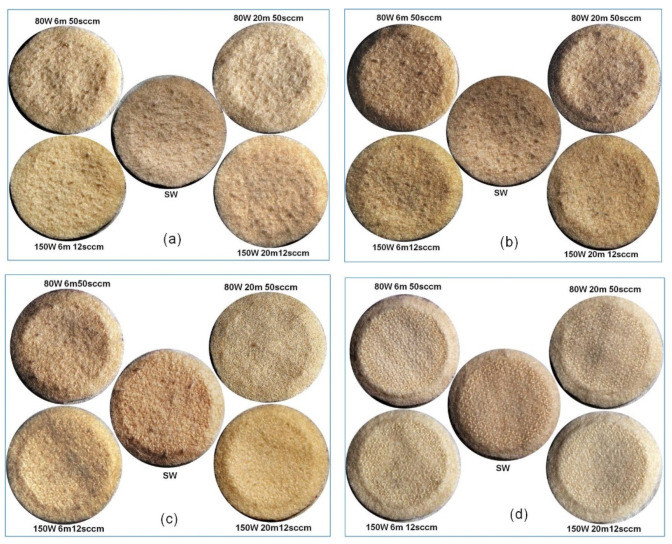
Effect of abrasion cycles on the surface damage of scoured wool fabrics: (**a**) 500 cycles, (**b**) 1500 cycles, (**c**) 3000 cycles, and (**d**) 10,000 cycles.

The visible damage on the surface of scoured wool fabrics after different numbers of abrasion cycles is presented in Figure 2. Previous studies have also characterised the visual appearance of fabrics after different numbers of abrasion cycles of Martindale testing by capturing photographs using either a digital camera [19] or SEM [20].

As can be seen in Figure 2, at low numbers of abrasion cycles, e.g., 500 cycles, the level of surface damage was low in all samples. Nevertheless, the lowest surface abrasion was observed in the case of C_2_F_6_ plasma-treated samples at 150 W, 20 min, 12 sccm. The samples treated at 80 W, 50 sccm (both 6 min and 20 min) showed inferior abrasion resistance to that of scoured wool and other plasma-treated samples. The surface damage of samples at 1500 abrasion cycles showed the same trend as at 500 cycles. At higher numbers of abrasion cycles, e.g., at 3000 cycles, the scoured wool sample showed the highest degree of surface damage. The samples treated with plasma at 150 W, 12 sccm showed superior abrasion resistance at both 6 min and 20 min, while the best result was achieved with 20 min treatment time, similar to the results for lower numbers of abrasion cycles. At 10,000 abrasion cycles, all samples exhibited significant noticeable surface damage. However, similar to at lower numbers of abrasion cycles, the highest abrasion resistance was observed in the case of plasma-treated samples at 150 W, 20 min, 12 sccm.

**Figure 3:**
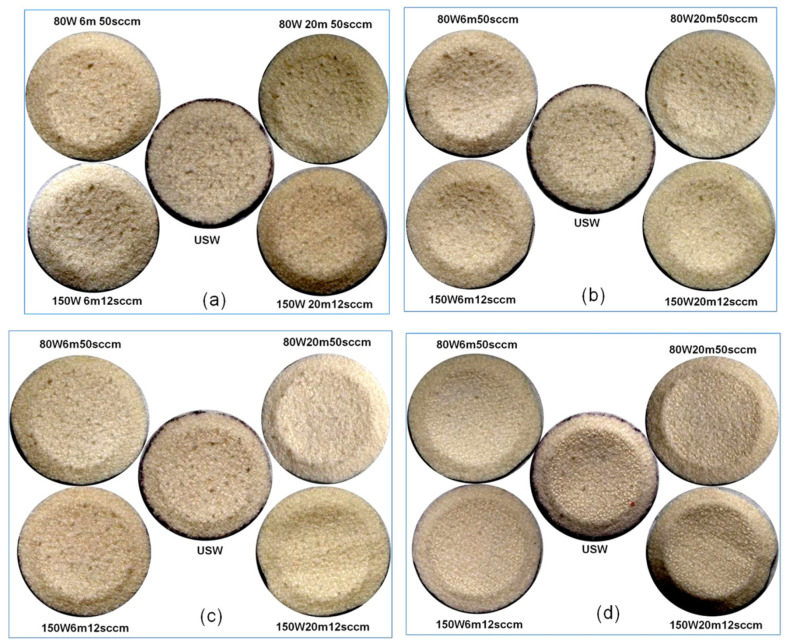
Effect of number of abrasion cycles on the surface damage of unscoured wool fabrics: (**a**) 500 cycles, (**b**) 1500 cycles, (**c**) 3000 cycles, and (**d**) 10,000 cycles.

Surface damage to the unscoured wool fabrics at different numbers of abrasion cycles also showed the same trend as the scoured wool fabrics, as shown in Figure 3.

**Figure 4:**
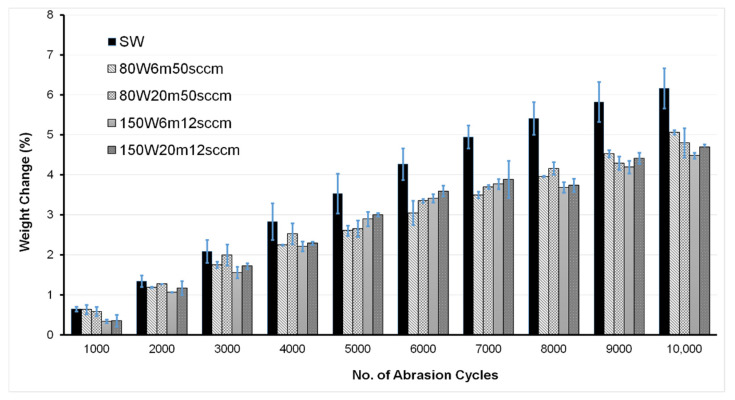
Weight loss of untreated and plasma-treated scoured wool fabrics at different numbers of abrasion cycles.

**Figure 5:**
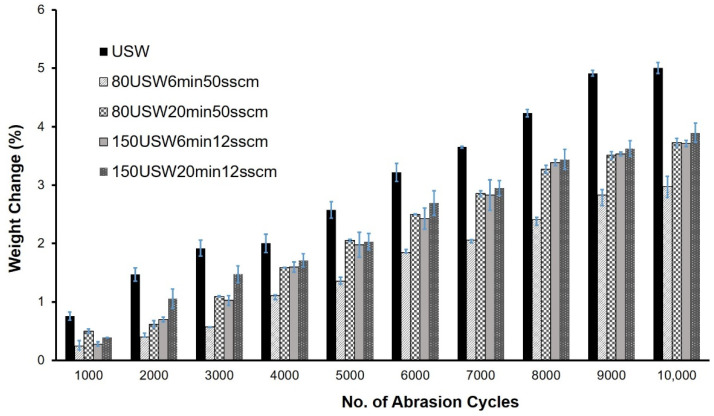
Weight loss of untreated and plasma-treated unscoured wool fabrics at different numbers of abrasion cycles.

Following the ISO 12947-3 standard for Martindale testing, the weight loss of fabrics after different numbers of abrasion cycles was calculated in order to quantitatively evaluate the abrasion damage in the fabrics. As can be seen in Figure 4, the weight loss results of the scoured wool fabrics at different numbers of abrasion cycles were also in agreement with the visual appearance of the samples. The weight loss of scoured wool fabrics increased with the number of abrasion cycles. As can be observed from Figure 5, a greater degree of weight loss was observed in the case of scoured wool samples at all abrasion cycles than in the unscoured samples, probably due to the presence of wax in the unscoured wool fibres, which acted as a lubricant, reducing the abrasion damage. Previous studies have also reported significant fabric weight loss after Martindale abrasion cycles in different textile fabrics [20]. Overall, the samples treated at 150 W, 12 sccm exhibited the lowest level of weight loss (especially at higher numbers of abrasion cycles), which was also evident from the visual appearance of the samples. The weight loss of the unscoured wool fabrics at different numbers of abrasion cycles also showed the same trend (except that the lowest weight loss in this case was achieved at 80 W, 6 min, 50 sccm), as can be observed from Figure 5. Further studies are underway to properly understand the difference in surface damage between different plasma-treated samples.

Improvement in the abrasion resistance of textile fabrics through surface modification and finishing has been noticed previously, and was mainly attributed to the application of hard coatings and a reduction in the frictional resistance of the fabrics [21,22,23,24].

For example, a hard coating of hybrid SiO_2_/Al_2_O_3_ sols onto cotton fabrics improved the abrasion resistance over 100,000 cycles in Martindale abrasion testing [21,22]. Similarly, the application of fluoropolymer coatings such as poly (vinylidene fluoride) was found to be effective in reducing the frictional properties of sports textiles [25]. This is the first study reporting a reduced weight loss during Martindale as a result of plasma treatment. The improved abrasion resistance of C_2_F_6_ plasma-treated unscoured and scoured wool fabrics obtained in this research could be due to two main possible reasons: (a) reduced roughness of fibre surface due to the plasma etching process [25,26], and (b) the presence of fluorine atoms on the wool fibre surface providing a lubricating effect [26,27]. In the case of C_2_F_6_ plasma, due to the high F/C ratio of the starting fluorocarbon, plasma-induced polymer vapour deposition on the wool fibre surface was not much expected, whereas plasma–fibre surface interaction could form pendant bonds through the removal of atoms and molecules, allowing the attachment of fluorine atoms [26,27]. Therefore, etching and surface grafting processes were expected to predominate over plasma polymerisation and deposition in the case of C_2_F_6_ plasma [26,27]. Previous studies on fluorocarbon plasma with high F/C ratios in the starting fluorocarbons, such as SF_6_, C_2_F_6_, etc., showed a significant reduction in surface roughness due to etching [25,26]. For example, Prestes et al. treated virgin and recycled PVC with SF_6_ plasma, and the surface roughness was measured using a 5-µm diameter diamond tip in a surface profiler [25]. It was observed that the SF_6_ plasma reduced the nano roughness of PVC, reducing the R_a_ values (i.e., the arithmetic average of the absolute values of the surface height deviations measured from the mean plane) of virgin and recycled PVC to 99.8 nm and 116.0 nm from 117.7 nm and 141.4 nm, respectively. Due to the inherently rough surface of wool fibres, plasma etching could be expected to be more effective on the hills than in the valleys of a nano roughness profile, thereby levelling the fibre surface [25]. As plasma etching increased with the plasma power and treatment time, the samples treated at 150 W for 20 min exhibited superior abrasion resistance. In contrast to this finding, plasma etching with C_2_F_6_ resulted in an increased roughness of lyocell fibres in a previous study [27]. In this study, fibre roughness was measured using atomic force microscopy (AFM), and the R_a_ values were compared. The roughness created by C_2_F_6_ plasma was on the nano level, with the R_a_ value of the untreated fibre increasing from 0.18 nm to 2.27 nm after 20 min of plasma treatment. This was attributed to the very smooth surface of untreated lyocell fibres (with a R_a_ value of only 0.18 nm), in which plasma etching resulted in an increased roughness, in contrast to natural fibres like cotton and wool, which possess relatively rough surfaces, whereby plasma etching can lead to the smoothening of the fibre surface, as observed in the present study. In the future, wool fibre surfaces can be further characterised using atomic force microscopy to quantitatively measure the roughness and to investigate whether surface levelling took place after the plasma treatment.

**Figure 6:**
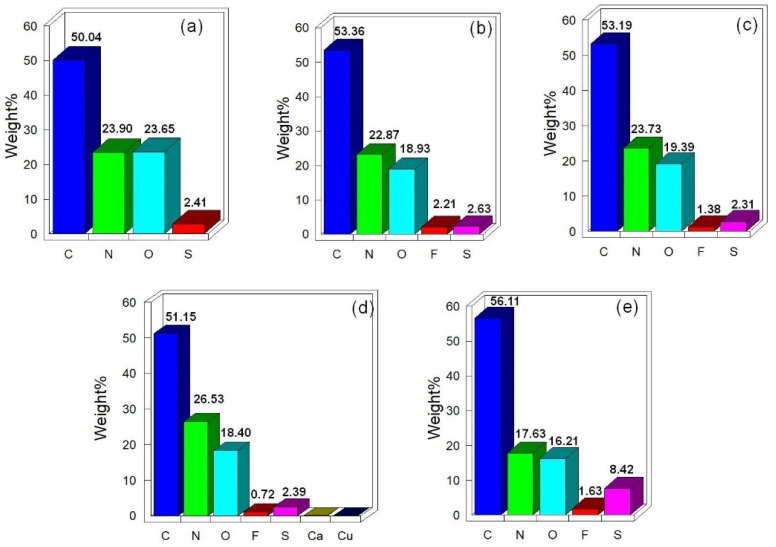
Relative wt. percentage of atoms in different scoured wool samples as characterised by EDX: (**a**) untreated, (**b**) 80 W 6 m 50 sccm, (**c**) 80 W 20 m 50 sccm, (**d**) 150 W 6 m 12 sccm, and (**e**) 150 W 20 m 12 sccm.

The presence of fluorine atoms on the surface of plasma-treated wool fibre could also induce a lubrication effect and improve the abrasion resistance of wool fabrics. Fluoropolymers are well known for their very low co-efficient of friction, and therefore, they have been widely used in coatings to improve the frictional and wear behaviour of different materials [28]. In fluoropolymers, due to the large size of fluorine atoms, they cover the carbon atoms in the polymer chains. Being highly electronegative, fluorine atoms thus shield the positive charge on the carbon atoms and the repulsion between the negative charges on adjacent fluorine atoms makes the cohesive force between the molecules very weak [29]. As a result, the molecules slip past each other very easily, leading to a very low co-efficient of friction [24]. In addition, very strong bonding between fluorine and carbon atoms results in no electron interaction with surrounding atoms leading to molecules with very low surface energy and enhanced lubrication property [29]. EDX study confirmed the presence of fluorine atoms on wool fibre surface, as shown in Figure 6.

It can be seen that the untreated unscoured wool fabrics mainly contained C, O, N and S, whereas a certain amount of F atoms can be observed in the EDX spectra of C_2_F_6_ plasma-treated wool fibres. The presence of C, O, N and S in the plasma-treated wool samples was attributed to chemical functional groups such as carboxylic acid (-COOH), amino (-NH_2_) groups, and disulphide crosslinks, which are present in the chemical structure of wool fibres. The presence of fluorine on plasma-treated wool fibres could be due to the reaction of F atoms and CF_x_ free radicals with the fibre surface [27], as well as significant deposition of C_x_F_y_ free radicals at higher treatment time (i.e., 20 min), as also evidenced in earlier studies from the presence of C_2_F_5_^−^, C_3_F_7_^−^, C_4_F_9_^−^, C_5_F_11_^−^ and C_6_F_13_^−^ on the lyocell fibre surface [27]. An increase in C wt.% in the plasma-treated samples (especially, in 150 W 20 m 12 sccm) also indicates the deposition of C_x_F_y_ free radicals. Further analysis will be carried out in future such as using X-ray photoelectron spectroscopy (XPS) [30] to characterise the chemical properties of plasma-treated wool fibre surface. As the amount of deposited fluorine compounds was low, it is expected to have a low environmental impact. However, further studies will be carried out in the future to investigate this.

It is well understood from the existing literature that C_2_F_6_ does not form a smooth and continuous fluoropolymer film on the wool fibre surface [26,27], which could be very effective in reducing surface energy as well as frictional properties. Nonetheless, the deposition of fluorine-containing molecules could potentially form a discontinuous film of lubricant in the boundary lubrication regime, allowing contact points between surfaces due to the discontinuity in the lubricant film [31]. Although the frictional coefficient in this case would be higher than the lubrication in the hydrodynamic regime (obtained in case of continuous film), it would be significantly lower than clean surfaces without any lubricant, i.e., wool surfaces without any fluorine-containing molecules [31]. The lubricating effect of fluorine and the reduction of frictional properties will be further confirmed in future by measuring the frictional co-efficient of untreated and plasma-treated wool fabrics.

### 3.2. Effect of Plasma Treatment on Contact Angle

**Figure 7:**
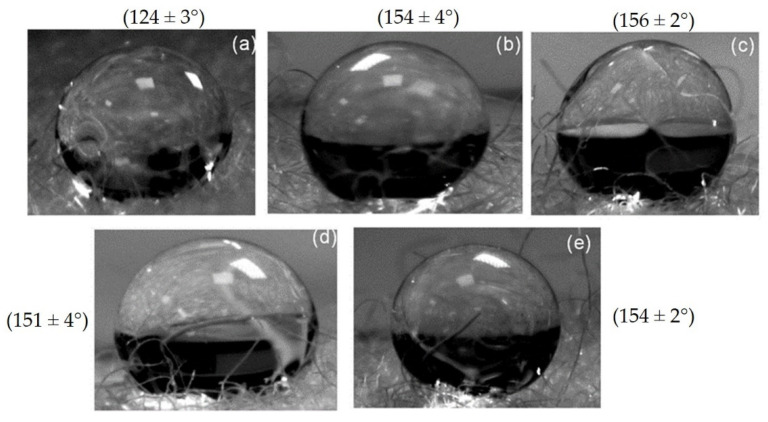
Water droplets on different scoured wool samples as captured by a digital camera: (**a**) untreated, (**b**) 80 W 6 m 50 sccm, (**c**) 80 W 20 m 50 sccm, (**d**) 150 W 6 m 12 sccm, and (**e**) 150 W 20 m 12 sccm (the contact angles are provided within brackets).

The contact angles of the scoured and plasma-treated wool fabrics under different conditions (the error values are based on 95% confidence intervals) as well as pictures of the water droplets caught (within 10 s) on wool fabrics, are shown in Figure 7. It can be noticed that the scoured wool sample shows a significantly lower contact angle (~124°) than those of the plasma-treated samples. The unscoured wool fibres were reported to be hydrophobic due to the presence of a covalently bound fatty acid, the chiral 18-methyleicosanoic acid (18-MEA), in the outer surface of the cuticle cells of wool fibres [32]. Although scouring of wool fibres led to the removal of most of the above fatty acids, the residual amount imparted a hydrophobic character to scoured wool fibres, and a contact angle to water of ~124° was achieved. However, this contact angle was not stable and started to decrease rapidly after 4 min 40 s, and the water droplet immediately spread over the wool fibre surface, as shown in Figure 7.

Several previous studies have attempted to measure the water contact angle of wool fibres, yarns and fabrics, with wide variability being obtained in the contact angle data, depending on the variability in the structural (i.e., fibre type, wax content, yarn and fabric structure, etc.) as well as measurement parameters (i.e., drop size, dropping height, etc.) [33]. The rough surface of wool fibres, yarns and fabrics has been reported to be the main difficulty in measuring the contact angle accurately. A similar contact angle value for untreated wool fabrics (~120°) to that obtained in the present study has also been reported previously [34].

**Figure 8:**
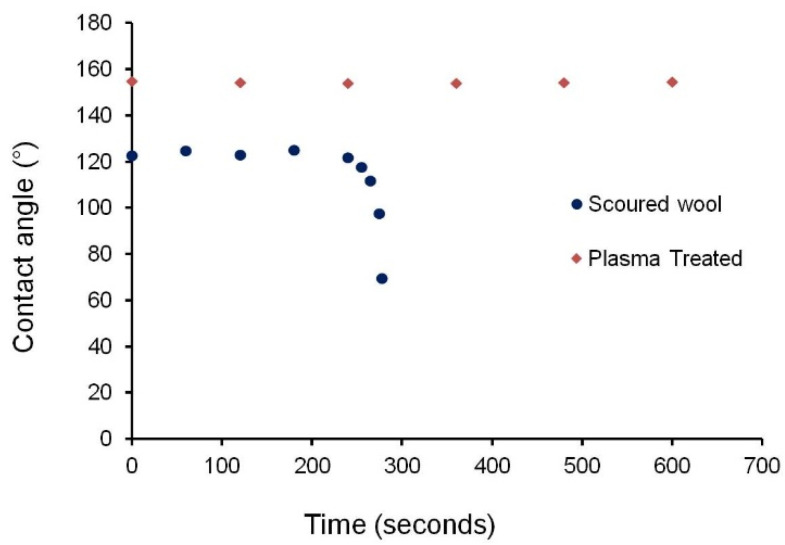
Change of contact angle with time for scoured and plasma-treated wool samples.

Due to the rough, heterogeneous, and porous surface of wool fabrics (and for fibres with similar characteristics), the Cassie-Baxter state of wetting, which assumes that a water droplet sits on top of the fibre surface and remains partially in contact with the entrapped air, was applied [35,36]. Figure 7 shows that the water droplets were mainly in contact with the loose fibres present on the surface of wool fabrics. On the other hand, the Wenzel state, which assumes that the water droplet penetrates the surface fibres and remains in full contact with the fibre surface, could not be applied for the present wool fabrics [34,35]. In the case of the scoured wool fabrics, the Cassie-Baxter state was not stable. The water droplet started to penetrate the structure, then contacted the whole fibre surface, and finally spread over the surface. The time taken for this process was 4 min 40 s, as can be seen in Figure 8.

It can be noted that all C_2_F_6_ plasma-treated samples exhibited contact angles higher than 150°, i.e., they showed super-hydrophobicity, and the contact angle was stable over a long period of time (see Figure 8). The plasma-treated sample at 150 W, 20 min, 12 sccm showed a constant contact angle over the studied 20 min period (the same trend was also achieved with other plasma-treated samples). The Cassie-Baxter state was stable in the case of plasma-treated samples, probably because the water droplet was not able to penetrate through the surface fibres owing to the hydrophobic character imparted by the C_2_F_6_ plasma treatment. No significant difference in the contact angle was observed between the different plasma-treated wool samples, indicating that the contact angle was largely dependent on the contact with the entrapped air and the level of hydrophobicity obtained in all plasma-treated samples was sufficient to maintain the Cassie-Baxter state. These findings suggest that C_2_F_6_ plasma treatment could produce a stable super-hydrophobic surface on wool fabrics.

### 3.3. Effect of Plasma Treatment on Dye Adsorption

The effect of C_2_F_6_ plasma treatment on the uptake of CI Acid Red 1 dye and K/S values of unscoured and scoured wool fabrics can be observed from Table 3. The decrease (%) in dye exhaustion and K/S values for plasma-treated unscoured and scoured fabrics were calculated by comparing with the values obtained for untreated unscoured and scoured fabrics, respectively.

It can be observed that C_2_F_6_ plasma treatment reduced the uptake of acid dye significantly in the case of both unscoured and scoured wool fabrics. This resulted in a corresponding change in the K/S values, as they were dependent on the concentration of the dye on the fibres [37]. Several previous studies have investigated the effect of different plasma treatments (such as helium, argon, oxygen, nitrogen, air, etc., but not C_2_F_6_) of wool fibres on their dyeing behaviours [32,38,39,40,41,42]. Most of these studies indicated a significant removal of the fatty acid layer from the wool fibre surface, which acted as the diffusion barrier for dye molecules. Removal of this hydrophobic layer due to plasma treatment exposed the proteinaceous layer, increasing the electrostatic interactions between wool fibres and dye molecules [38]. Consequently, the rate of dyeing of wool fibres with different dyestuff (mainly hydrophilic dyes) increased due to plasma treatment [38]. An increase in new amino groups on the wool fibre surface due to nitrogen plasma treatment also led to increased dyeing rates [40]. In addition to that, cystine oxidation in the A layer of exocuticle of wool fibres was also noticed as a result of plasma treatment [38]. This resulted in the decreased concentration of disulphide crosslinks, facilitating transcellular and intercellular dye diffusion within wool fibre structure [38]. The observed increased dye exhaustion because of plasma treatment could be attributed to this phenomenon. A few researchers have also noticed that plasma treatment changed the physical structure of wool fibres [32,42]. Mainly, significant changes in the scale structure were observed. The well-defined edges of scales in the untreated wool fibres became eroded, non-uniform and open due to plasma etching and this could also facilitate the diffusion of dye molecules into the wool fibre structure [32,42].

**Figure 9:**
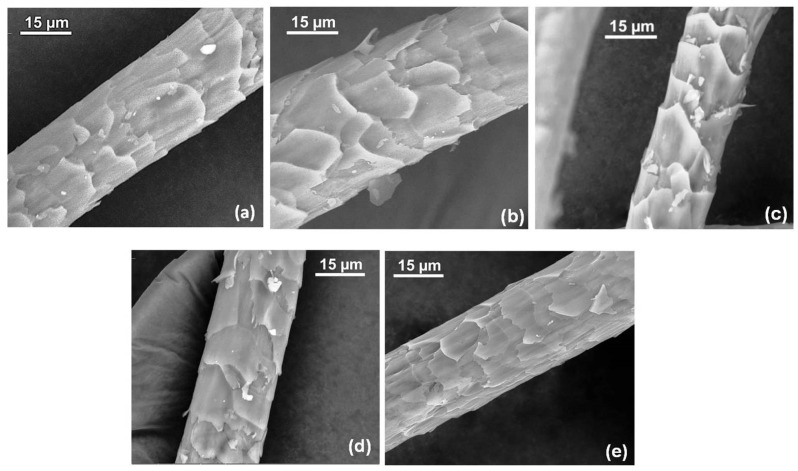
Scale morphology of unscoured wool fibres: (**a**) untreated, (**b**) 80 W 6 m 50 sccm, (**c**) 80 W 20 m 50 sccm, (**d**) 150 W 6 m 12 sccm, and (**e**) 150 W 20 m 12 sccm.

In contrast to previous studies, the C_2_F_6_ plasma treatment in the present study resulted in an increased hydrophobicity of the wool fibre surface due to the presence of fluorine-containing molecules, as discussed in Section 3.1, and was confirmed from the higher and stable water contact angle in the case of plasma-treated wool fabrics. These hydrophobic elements acted as a barrier to dye diffusion on the wool fibre surface (i.e., increased the diffusion boundary layer), resulting in decreased dye exhaustion and colour strengths. However, as also noticed in other studies, the change in dye exhaustion due to plasma treatment was limited due to dyeing at a high temperature (i.e., 100 °C). The high-temperature dyeing process facilitated the diffusion of dye molecules both from the dye bath to fibre surface and from fibre surface to the interior, thus improving the equilibrium exhaustion of the plasma-treated samples. In addition to this, the high-temperature dyeing process was also able to remove the hydrophobic elements from the wool fibre surface to some extent, reducing the effect of C_2_F_6_ plasma treatment on the dye exhaustion. Dyeing at lower temperature could lead to a higher difference in exhaustion values, as previously observed in case of other types of plasma treatments of wool fibres [38]. Another reason behind the low impact of C_2_F_6_ plasma treatment on the dye exhaustion could be the improved dye diffusion through the eroded and open scale structure of plasma-treated fibres, which acted as an opposing factor to the effect of hydrophobicity on the dyeing behaviour. Figure 9 shows the scale morphology of untreated and plasma-treated wool fibres. The scales in the untreated fibres (Figure 9a) were relatively more uniform and overlapped compactly, whereas the scales in the plasma-treated samples were mostly eroded, creating broken edges and open spaces for dye diffusion in the fibre structure, as shown in Figure 9b–e.

Among the different plasma-treated samples, the highest reduction in the dye exhaustion and K/S values over untreated samples were achieved in the case of the sample treated with 150 W 20 min 20 sccm in both unscoured and scoured wool fibres. These samples also showed superior performance in the case of abrasion resistance, indicating deposition of a higher amount of fluorine-containing molecules. The plasma-treated samples at 150 W, 6 min, 20 sccm (i.e., with same power and flow rate, but at a reduced treatment time) led to the lowest change in dye exhaustion and K/S values, indicating the significant influence of plasma treatment time on dye adsorption. However, the effect of treatment time was exactly the opposite in the case of 80 W and 50 sccm, where an increase in the treatment time from 6 min to 20 min reduced the influence of C_2_F_6_ plasma treatment. Further investigation is underway to understand this phenomenon. It can also be noticed that similar dye exhaustion values were obtained in the case of samples treated at 150 W, 20 min, 12 sccm and 80 W, 6 min, 50 sccm, indicating that a similar effect on dyeing behaviour could be achieved at a lower power and with a shorter treatment time by increasing the gas flow rate to 50 sccm.

## 4. Conclusions

In the present study, wool fabrics were treated with C_2_F_6_ gas while varying plasma power (80 W and 150 W), gas flow rate (12 sccm and 20 sccm) and treatment time (6 min and 20 min), and were subsequently characterised for abrasion resistance, contact angle and dyeing behaviour. The following conclusions can be made from this study:

(a) C_2_F_6_ plasma treatment had significant influence on the abrasion resistance of both unscoured and scoured wool fabrics. Both visual surface damage and weight loss of wool fabrics increased with the increase in number of abrasion cycles from 500 to 10,000 cycles. The untreated wool fabrics showed the highest surface damage and weight loss among all of the samples, whereas the fabrics treated at 150 W, 12 min, 20 sccm showed the lowest visible surface damage.
(b) Plasma treatment of wool fabrics showed (from the EDX study) the presence of F atoms, along with C, O, N and S atoms (which were also observed in the untreated wool fabrics), and an increase in C wt.%. This could be attributed to the reaction of F atoms and CF_x_ free radicals with the wool fibre surface, as well as significant deposition of C_x_F_y_ free radicals.
(c) The plasma treatment imparted superhydrophobicity (i.e., contact angles higher than 150°) to the wool fabrics. The untreated wool fabrics showed significantly lower contact angles (~124°), which were not stable. On the contrary, the plasma-treated samples showed a stable contact angle due to their rough fabric structure, with loose surface fibres and increased hydrophobicity.
(d) The acid dye exhaustion of the wool fabrics decreased significantly due to the increased hydrophobicity of the wool fabrics owing to plasma treatment. The highest effect was observed in the case of samples treated at 150 W, 12 min, 12 sccm.

## Figures and Tables

**Table 1:** Properties of Acid Red 1 dye.

Parameters	Value *
Chemical Name	Amido Naphthol Red G, Azophloxine
Formula Weight	509.42
Charge	negative
Molecular Formula	C_18_H_13_N_3_Na_2_O_8_S_2_
Chromophore	Mono azo
Maximum Wavelength	$\lambda_{max}=506\;\mathrm{nm}$, $\lambda_{max}=532\;\mathrm{nm}$ (2nd)

* Manufacturer data.

**Table 2:** Plasma treatment conditions of wool fabrics.

Sample Codes	Plasma Conditions		
	Power (W)	Flow Rate (Sccm)	Time (min)
Un-scoured (USW) and scoured wool (SW) fabrics	-	-	-
80 W 6 m 50 sccm	80	50	6
80 W 20 m 50 sccm	80	50	20
150 W 6 m 12 sccm	150	12	6
150 W 20 m 12 sccm	150	12	20

**Table 3:** Dye uptake and K/S values of untreated and plasma-treated wool fabrics.

Samples	Dye Exhaustion	% Decrease in Dye Uptake	K/S	% Decrease in K/S
USW	96.67	---	41.13 ± 0.88 *	-----
80 W 6 m 50 sscm	88.44	8.5	35.25 ± 0.83	14.3
80 W 20 m 50 sscm	89.44	7.5	36.00 ± 0.77	12.5
150 W 6 m 12 sscm	91.66	5.2	40.36 ± 0.48	1.9
150 W 20 m 12 sccm	87.77	9.2	34.13 ± 1.49	17.0
SW	96.70	----	39.13 ±0.87	-----
80 W 6 m 50 sscm	88.67	8.3	35.75 ± 1.08	8.6
80 W 20 m 50 sscm	89.99	6.9	36.89 ± 0.51	5.7
150 W 6 m 12 sscm	92.22	4.6	40.38 ± 0.69	3.2
150 W 20 m 12 sscm	87.77	9.2	34.63 ± 1.43	11.5

* The error values are based on 95% confidence intervals.

## Data Availability

The data that support the findings of this study are available on request from the corresponding author. The data are not publicly available due to privacy or ethical restrictions.
